# Planar Zeolite Film-Based Potentiometric Gas Sensors Manufactured by a Combined Thick-Film and Electroplating Technique

**DOI:** 10.3390/s110807736

**Published:** 2011-08-05

**Authors:** Isabella Marr, Sebastian Reiß, Gunter Hagen, Ralf Moos

**Affiliations:** Department of Functional Materials, University of Bayreuth, Bayreuth 95440, Germany; E-Mail: Functional.Materials@Uni-Bayreuth.de

**Keywords:** solid state hydrocarbon gas sensor, zeolite, MFI, ZSM-5

## Abstract

Zeolites are promising materials in the field of gas sensors. In this technology-oriented paper, a planar setup for potentiometric hydrocarbon and hydrogen gas sensors using zeolites as ionic sodium conductors is presented, in which the Pt-loaded Na-ZSM-5 zeolite is applied using a thick-film technique between two interdigitated gold electrodes and one of them is selectively covered for the first time by an electroplated chromium oxide film. The influence of the sensor temperature, the type of hydrocarbons, the zeolite film thickness, and the chromium oxide film thickness is investigated. The influence of the zeolite on the sensor response is briefly discussed in the light of studies dealing with zeolites as selectivity-enhancing cover layers.

## 1. Introduction

Zeolites are microporous, crystalline aluminosilicate materials with an enormous inner surface. Their special framework structure and their associated unique physicochemical properties make them an interesting class of materials that is used for catalysts [1], ion exchangers [2], and gas adsorbers, for instance, in automotive exhaust gas aftertreatment applications [3]. Zeolites are composed of [SiO_4_]^4−^ and [AlO_4_]^5−^ tetrahedra with the metal ion in the centre being linked to oxygen corners. The pure silicate framework is uncharged, but an aluminosilicate framework comprises one negative charge per [AlO_4_]^5−^ tetrahedron [4]. To compensate the negative charge, mobile exchangeable cations, e.g., Na^+^, K^+^ or Ca^2+^, are incorporated into the zeolite lattice. The general chemical formula of zeolites is:

(1) Aymm+⌊(SiO2)x⋅(AlO2−)y⌋⋅zH2O

Here, A is a mobile and exchangeable cation with the charge *m*, *x* + *y* is the number of tetrahedra per crystallographic unit cell, and *x*/*y* is the Si/Al ratio of the framework [1]. The Löwenstein rule reveals that *x*/*y* ≥ 1, meaning that Al-O-Al bonds are forbidden and only Si-O-Si or Si-O-Al bonds are allowed [5]. The tetrahedra are linked to larger composite building units, like the sodalite cage or the pentasil unit [6]. The latter is the characteristic composite building units of the zeolite type ZSM-5, which is used in its sodium modification in our work. Pentasil units are built up to chains that establish the framework structure of ZSM-5. The two-dimensional pore system is connected by zigzag channels [7]. The diameter of the channels is 0.56 × 0.53 nm and 0.55 × 0.51 nm [1].

Some recent applications of zeolite films and membranes are reviewed in [8]. Compared with semiconducting oxides, zeolites have rarely been investigated for gas sensing purposes. Recent reviews [9–11] point out that zeolites are either used as filters (more generally described as “auxiliary elements” [12]) or as functional elements for sensor principles relying directly on the conductive, adsorptive, or catalytic properties of the zeolite, triggered by the zeolite’s interaction with the surrounding atmosphere. In some pronounced applications, a selective adsorption of an analyte changes the electrical impedance of the zeolite. This principle can be utilized, for instance, for hydrocarbon gas sensing [13–15], for ammonia gas sensing [16], or for water vapor detection [17,18]. Usually, the sensors are manufactured in a planar technology with the zeolites being screen-printed onto interdigitated electrodes (abbreviated as IDE in what follows) that have been previously applied on insulating alumina substrates. The typical operation temperature is between 200 and 500 °C. The advantage of the planar setup is a fast diffusion of the analyte through the zeolite with the result of a fast sensor response. In the case of the ammonia sensor, it is obvious that the bulk properties change with ammonia adsorption [19], whereas in some hydrocarbon sensors, electrode/bulk interactions seem to play a major role [20]. The detailed effect remains a subject of discussion [21,22].

Besides the impedimetric principle, a potentiometric setup, where a potential difference, *U*, between two electrodes is the measurand, has been suggested for hydrocarbon sensing [23] in a recent approach. It comprises bulk-type plane-parallel polished zeolite discs with a gold electrode on one side and a solid-state reference electrode of Na_0.85_CoO_2_ that provides a constant sodium activity on the other side [24]. A disc of BaCO_3_/Na_2_CO_3_ composite (after [25]) served as a Na^+^ ion bridge to seal the reference electrode hermetically from the surrounding gas phase. The setup shows a good hydrocarbon sensitivity but is cross-sensitive to CO_2_ and O_2_ (as a result of the sodium reference). In addition, the bulk-type setup with the sodium reference is too complicated from an application-oriented point of view. As shown in [26], the bulk-type setup with solid-state reference electrode of Na_0.85_CoO_2_ shows a semilogarithmic behavior of the sensor signal *U vs.* propane concentration, however, only in the percent range. The origin of the formation of the potential difference *U* between both electrodes is under study and will explicitly not be discussed here. The interested reader is referred to the literature [21,22,27].

Very recently, a planar *potentiometric* zeolite-based gas sensor has been described [28]. In this very initial study, one of the two gold electrodes is selectively covered by evaporation with different metal oxides with a thickness of only a few nm. The electrodes are covered with a thick-film of Pt-loaded zeolite ZSM-5. Different sensor configurations have been measured and almost all of them show a hydrogen sensitivity. Some of the sensors show a cross-sensitivity towards hydrocarbons, which was exemplarily shown for butane as a representative hydrocarbon.

The purpose of the present work is to transfer the sensor setup of [28] into a vacuum technique-free low-cost production technology to produce hydrocarbon or hydrogen gas sensing devices. This work should also be seen as an explorative approach to study the parameters that affect the sensor response and the cross-sensitivity. Especially, the sensor response towards different hydrocarbons will be investigated in detail. At the end, the influence of the zeolite on the sensor parameters is briefly discussed and compared with literature data.

## 2. Experimental

**Figure 1.**
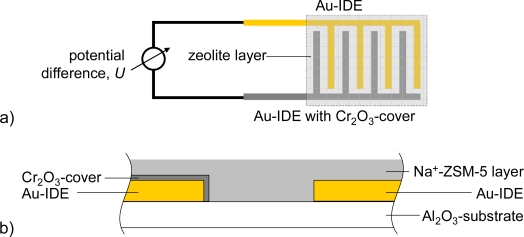
**(a)** Top view of the sensor; **(b)** Cross section of the sensor. IDE is an abbreviation for interdigital electrodes.

The schematic sensor setup is depicted in Figure 1. On an alumina substrate (96% Al_2_O_3_ Rubalit 708S, CeramTec) two interdigitated Au electrodes were screen-printed (paste KQ500, Heraeus). The line and space widths of the electrode fingers were 100 μm, the thickness of the gold thick-film electrodes was 5 μm in average. The transducers were heat treated in air at 850 °C for 15 min.

**Figure 2.**
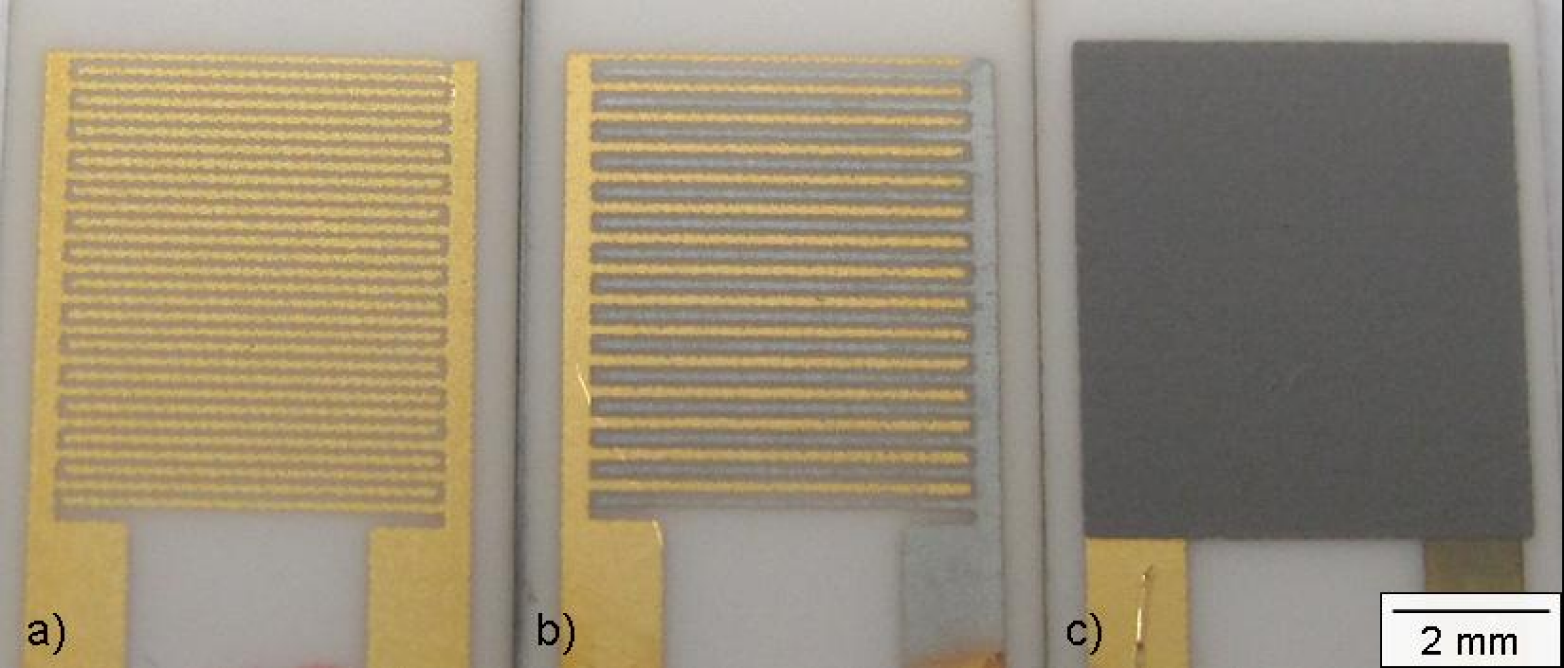
**(a)** Screen-printed and sintered interdigital gold electrodes; **(b)** One selectively electroplated Cr-covered gold electrode; **(c)** Entire setup with screen-printed zeolite-layer.

Figure 2(a) shows a transducer after firing. Then, one interdigital Au electrode of each IDE was Cr electroplated with Pb as a counter electrode. Impedimetric zeolite-based gas sensors with one chromed interdigital electrode have recently been presented [29]. The procedure in this present work adopts this electroplating. The transducers were dipped into a Cr containing solution (Glanzchrombad CR 843, Atotech), covering the interdigital electrode completely. One electrode was connected to a constant current source (Keithley SourceMeter 2400) to apply an electric current. The interdigital electrode acts as the cathode, the Pb electrode as the anode. The electroplating process was operated at room temperature for 40 s with varied currents (currents decreased during the process to final values noted below and in the figures).

For the covers generated with different currents, one observed a change in the coloring, a first hint for different Cr film thicknesses. The thickness of the Cr cover increased with the current. From the coloring, it is estimated that Cr films prepared with the same current during the electroplating process have the same thickness [Figure 2(b)]. Table 1 displays the current, the corresponding coloring of the Cr covering and the estimated thicknesses of the Cr and the Cr_2_O_3_ cover. The evaluation of the thickness of the Cr respectively Cr_2_O_3_ cover was not successful by SEM imaging, probably due to the very thin Cr_2_O_3_ layer. However, an estimation can be given by calculating the thickness of the Cr layer with respect to the transferred electrons *n*_e_ during the plating process. According to Equations (2), *n*_e_ corresponds to the charge (current *I* for the time *t*) divided by the elementary charge *e*:

(2) $$n_{e}=\frac{I\cdot t}{e}$$

The chromium layer is assumed to be a dense layer and the thickness can be deduced from the molar volume *V*_m,Cr_. By Equation (3), the Cr layer thickness *d*_Cr_ is defined as the Cr volume divided by the IDE surface area *A*. If one assumes six transferred electrons per Cr atom on the metal surface, *d*_Cr_ can then be calculated from Equation (3):

(3) $$d_{\text{Cr}}=\frac{V_{\text{Cr}}}{A}=\frac{n_{e}\cdot V_{m,\text{Cr}}}{6\cdot N_{A}\cdot A}$$

By oxidizing the chromium layer to Cr_2_O_3_, the molar volume changes. Hence, the factor *z* is introduced according to Equation (4) to calculate the change in the volume and the layer thickness by the cube root. An additional factor *a* is experimentally identified as an efficiency factor for the process. This factor is measured by a plating time of 120 s. The resulting chromium layer can be measured and compared to the calculated thickness *d*_Cr_. The outcome of this is an efficiency factor of *a* = 0.4073.

(4) $$z=\sqrt[{3}]{\frac{V_{m,\text{Cr2O3}}}{2\cdot V_{m,\text{Cr}}}}\approx 1.263$$

(5) $$d=a\cdot z\cdot d_{\text{Cr}}$$

Thus, the Cr_2_O_3_ layer thickness can be estimated according to Equation (5) and is presented for the different current levels in Table 1.

Reasons for the decreasing current during the galvanic process may be found on the Pb counter electrode, in the Cr containing solution and in the limit of the voltage. After a few plating processes, the Pb electrode showed a yellowish layer on the surface. This coating could have had a passivation effect, meaning that the number of electrons that is emitted by the Pb counter electrode is reduced. Also, the number of Cr^6+^ ions decreased during the electroplating. Consequently, the transport of Cr ions in the solution would have been hindered. In any case, the voltage limit of the current source had limited the current. If the voltage was too high for the preset current of 30 mA, the constant current source would have lowered the current due to the voltage limit. Therefore, the thicknesses of the layers may be smaller than the calculated values in Table 1, which should be considered only as a maximum estimation.

The Pt loaded zeolites (1, 2, and 3 wt% Pt) were prepared with Na^+^-ZSM-5 zeolite powder (Süd-Chemie, SN-27, SiO_2_/Al_2_O_3_ ratio = 25,) by ion exchange in an aqueous solution of tetraammineplatinum(II) chloride (56.4 wt% Pt, Alfa Aesar). The suspension was stirred at room temperature for 24 h. After filtration, the residue was washed with water until no AgCl could be obtained after adding AgNO_3_ solution to the filtrated acidulated water. The Pt exchanged zeolite was dried for 12 h at 120 °C (after [27]).

Afterwards, the zeolite was reduced, either by NaBH_4_ or hydrogen (5% H_2_ in N_2_) according to [27] and [30], respectively. For the first reduction route, NaBH_4_ (Merck) was dissolved in water before the Pt loaded zeolite was added while stirring. After stirring for 24 h at room temperature, the zeolite powder was filtered, washed with water and dried at 80 °C for 12 h. The second way of reduction was carried out in a vertical furnace. The ion exchanged zeolite powder was heated to a maximum of 450 °C (heating/cooling rate: 10 °C/min) in a reducing gas atmosphere. The type of reduction influenced the species of mobile cations in the channel system of the zeolite and therefore the ionic conductivity. Reduction with NaBH_4_ leads to preservation of Na^+^, reduction in hydrogen leads to certain exchange of Na^+^ to H^+^ in the channels [30]. Therefore, the reduction method influences the ionic conductivity, which increases with higher sodium content. As the inner resistance of the ion conducting zeolite phase should be low to avoid noise in the voltage measurement, we tried both methods. From a catalytic point of view, the H^+^ containing zeolites should provide better properties.

**Figure 3.**
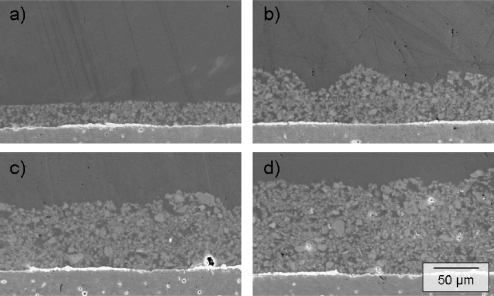
SEM micrographs of the cross section of a sensor with **(a)** one (∼25 μm); **(b)** two (∼50 μm); **(c)** three (∼75 μm); and **(d)** four (∼100 μm) screen-printed zeolite layers on gold interdigital electrodes on alumina substrates.

The reduced zeolite powders were processed to screen-printable pastes by mixing them with organic binders (KD 2721, Zschimmer & Schwarz) and homogenizing the mixture in a roller mill. The zeolite pastes were screen-printed on the Au/AuCr interdigital electrodes. To increase the thickness of the zeolite cover (one layer equals ∼25 μm), the transducers were dried for 5 min at 110 °C. Then, another layer of the zeolite paste was printed. The pastes were annealed for 6 h at 450 °C (heating rate: 0.8 °C/min). During the heat treatment, the organic compounds were removed and the Cr covers on the interdigital gold electrodes were oxidized to Cr(III) oxide. A picture of a final sensor is shown in Figure 2(c). The SEM micrographs (Figure 3) show cross sections from zeolite layers of different thicknesses. The thickness increases almost linearly with the number of screen-printed layers. The Cr(III) oxide layer on the opposite fingers of the interdigital electrode is not observable with this analysis method.

By this sensor preparation process, a wide variety of sensor setups could have been realized as shown in the modification matrix in Table 2.

**Figure 4.**
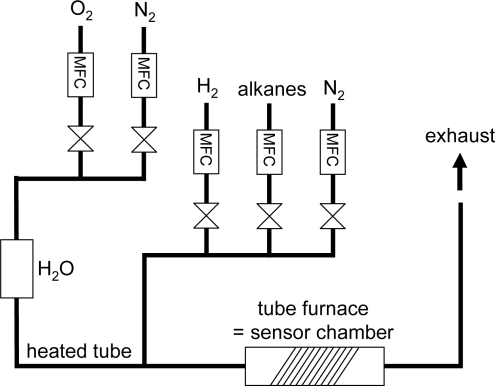
Setup of the gas sensor test bench.

For the sensor measurements, the base gas atmosphere was composed of 10% O_2_ and 2.5% H_2_O in an N_2_ flow. 500 and/or 1,000 ppm of the test gases H_2_, CH_4_, C_2_H_6_, C_3_H_8_, or n-C_4_H_10_ were added. The total gas flow was 600 mL/min. The sensor output is the potential difference, *U*, between both electrodes. Some authors denote this also as an electromotive force (*emf*). The potential difference *U* was measured with a digital multimeter (Keithley 2700, input resistance > 10 GΩ) in a tube furnace (the setup is described in Figure 4), in which the gas sensors were heated to 300, 350, or 400 °C. Further details of the test setup are given in [31]. The gas exchange time of this test bench is about 75 s. To compare the results of different experiments, the sensor response Δ*U*, which is defined as the difference of *U* in the base gas atmosphere and *U* in test gas atmosphere is plotted.

## 3. Results and Discussion

### 3.1. Influence of the Platinum Loading and of the Sensor Temperature

Initial tests were carried out to determine an optimum sensor temperature and Pt content. Sensors for that purpose were covered with one zeolite layer. Zeolites were loaded with 1, 2, and 3 wt% Pt and all were reduced in hydrogen. The applied temperatures were 300, 350, and 400 °C.

**Figure 5.**
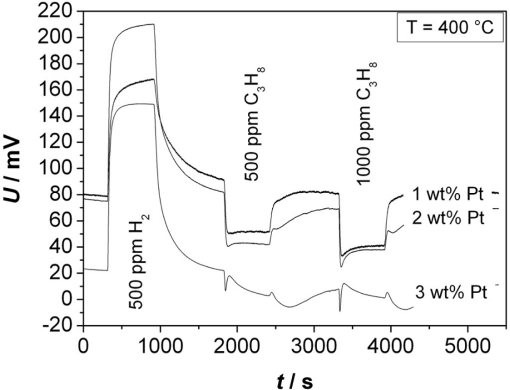
Potential difference *U* of sensors to H_2_ and C_3_H_8_ with differently platinum-loaded zeolites at 400 °C.

The measurement shown exemplarily in Figure 5 was conducted at 400 °C. It shows the response of a sensor toward H_2_ (500 ppm) and C_3_H_8_ (500 and 1,000 ppm). An obvious effect is that the potential difference *U* increases with H_2_, which is in contrast to C_3_H_8_ exposition that yields an opposite effect.

**Figure 6.**
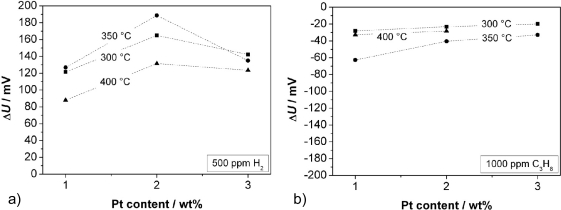
Sensor output change Δ*U* for differently platinum-loaded zeolites when exposed to **(a)** 500 ppm H_2_ and to **(b)** 1,000 ppm C_3_H_8_. Please note the opposite sign in figure (b).

Figure 6 shows the response to 500 ppm H_2_ (a) and to 1,000 ppm C_3_H_8_ (b). Independent from the Pt content, one observes the maximum change of Δ*U* at 350 °C, except for the measurement with 3 wt% Pt. In this case we assume such high catalytic activity that no temperature influence is observed. For a Pt content of 2 wt%, the sensors show—not depending on the temperature—the highest Δ*U* to hydrogen. In contrast, the signal to propane decreases with a higher Pt loading. The response to H_2_ is significantly higher than the response to C_3_H_8_. The faster diffusion and higher reactivity of hydrogen compared to propane may be a hint for the explanation of the sensing mechanism. The response time, *t*_90_, towards hydrogen was between 60 and 200 s, and towards propane between 100 and 500 s. At a temperature of 350 °C, *t*_90_ for H_2_ decreases with increasing Pt content. No further trends could be observed for the response time. In the next step, the influence of the sensor response on the chain length of admixed hydrocarbons was investigated.

**Figure 7.**
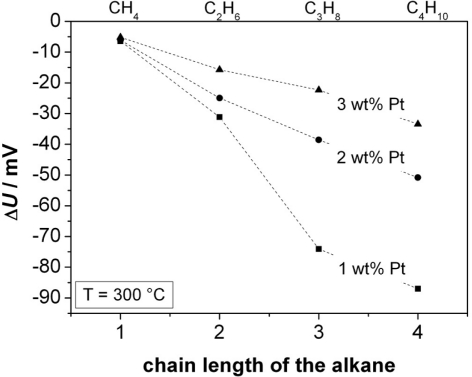
Sensor output change Δ*U* as a function of hydrocarbon chain length and Pt loading of the zeolite.

CH_4_, C_2_H_6_, C_3_H_8_, and n-C_4_H_10_, each of them in a concentration of 500 ppm, were added to the base gas separately. The sensor response at 300 °C is plotted in Figure 7 in dependence of the chain length of the alkanes for different Pt contents of the zeolite. One can observe that both a lower Pt loading and a higher C-atom number leads to a higher sensor response.

### 3.2. Influence of the Chromium Oxide Layer Thickness

**Figure 8.**
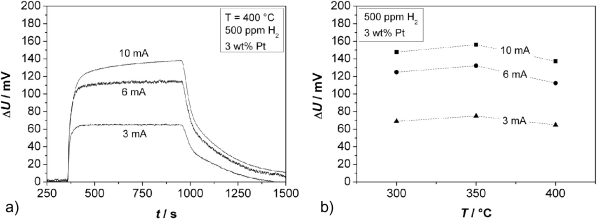
**(a)** Sensor response Δ*U* to an addition of 500 ppm H_2_ to the base gas at 400 °C; **(b)** Dependence of Δ*U* upon temperature and Cr_2_O_3_ film thickness.

Further experiments were conducted to determine the effect of the thickness of the thin Cr_2_O_3_ film covering one electrode. Sensors in accordance to Figure 1(b), Cr-electroplated with currents of 3, 6, and 10 mA, respectively, were tested. The zeolite film layer was made of a 3 wt% Pt loaded zeolite powder, reduced by NaBH_4_. Typical response curves towards 500 ppm H_2_ added to the base gas are shown in Figure 8(a) (for 400 °C) for the three different oxide film thicknesses.

**Figure 9.**
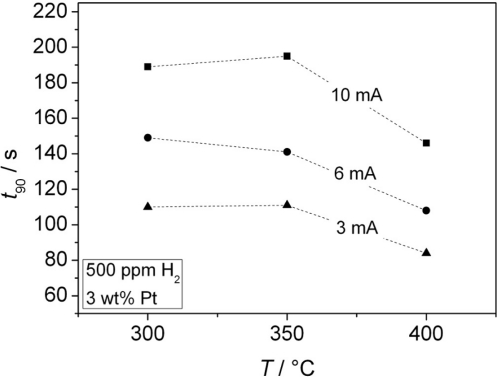
Dependence of the response time upon temperature and Cr_2_O_3_ film thickness.

From these curves, the sensor response time and the sensor output change Δ*U* [Figure 8(b)] was determined. The sensor responds faster for thinner chromium oxide layers (*t*_90_ = 84 s for the thinnest oxide layer at 400 °C, which is close to the gas exchange time of the test bench). The response time *t*_90_ of the sensors with different thicknesses of the Cr_2_O_3_ film is shown in dependence of the temperature in Figure 9.

The thickest layer, obtained with a galvanic current of 10 mA, does not even reach a final constant voltage. In contrast to the response time, a thicker oxide film increases the sensor signal. From the shape of the sensor response signal from Figure 8(a), one may assume that a second effect occurs at higher film thicknesses. It is astonishing that the sensor output *U* is by far more affected by the oxide film thickness than by the sensor temperature.

### 3.3. Influence of the Thickness of the Zeolite Layer

**Figure 10.**
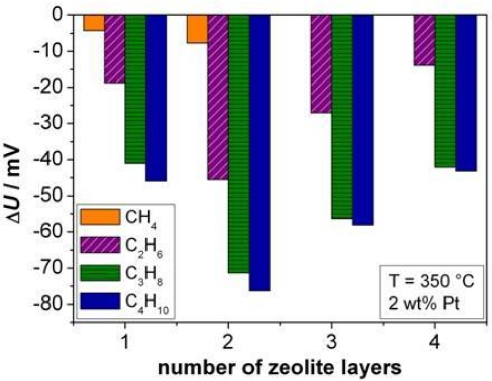
Δ*U* for different alkanes in dependence of the number of zeolite layers.

On sensors covered with different numbers of zeolite layers, the influence of the thickness of the zeolite layer was investigated, exemplarily shown at 350 °C. Since Figure 6 revealed the highest sensitivity for 2 wt% Pt loaded samples, the zeolite powders were loaded with this Pt amount and were reduced in hydrogen. The results are summarized in Figure 10.

The highest sensor output change Δ*U* was obtained for two zeolite layers (approximately 50 μm) for all alkanes. Again, the dependency of Δ*U* upon the alkane chain length can be observed. The response time towards C_2_H_6_ shows a similar trend as the sensor signal. The minimum *t*_90_ was found for two layers of zeolite with 124 s. For all other alkanes no certain trend could be observed. For methane, the response was very slow (about 500 s), for C_3_H_8_ and for n-C_4_H_10_, the response time was between 160 s and 260 s. These results agree to some extent with careful investigations of ZSM-5 zeolite filter layers, applied on top of conductometric sensors. In reference [32], the effect of Pt loaded zeolite cover layers has been quantitatively explained by a diffusion-reaction model, which states that hydrocarbons need to diffuse through the zeolite pores and get oxidized during the passage through the catalytically active zeolites. Furthermore it had been found out, that the response time *t*_90_ of the sensor increases with an increased cover layer thickness, in other words, the porous cover layer can be considered as a diffusion barrier. For cover layer thicknesses above 50 μm, a strong increase of the sensor response time had been found, leading to sensor response times in the range of over 210 s. Based on the results of reference [32], we can assume that during the diffusion through the zeolite layer, the concentration of the analyte gets reduced. This may explain the decrease of the sensor signal for more than two layers, as shown in Figure 10 (this effect is especially visible for CH_4_; this compound could not even be detected at 350 °C by a sensor covered with three zeolite layers containing 2 wt% Pt).

At first glance, it seems strange that the sensor signal increases with an increasing zeolite layer thickness (from one to two layers). However, this agrees with findings in reference [33], also for conductometric sensors. There, the response to 500 ppm saturated hydrocarbons as well as to 1,000 ppm H_2_ increases with a catalytically activated Pt loaded ZSM-5 filter layer. This is exactly what is observed here. One may speculate that during the diffusion through the zeolite a partial oxidation reaction occurs, leading to very reactive intermediate species. In the case of methane, a thickness of three layers might be sufficient to oxidize all molecules that diffuse through the zeolite layer to the electrode.

## 4. Conclusions and Outlook

It is demonstrated that a potentiometric hydrocarbon gas sensor using a sodium ion conductor and two different interdigital electrodes can be fully manufactured using a low-cost production technology involving thick-film and electroplating techniques. As shown in this exploratory approach, the sensor output can be modified by several parameters, like Pt loading of the zeolite, thickness of the Cr_2_O_3_ cover or thickness of the zeolite layer. The sensor output signal shows a maximum for a zeolite film thickness of about 50 μm (two layers). This effect has not been fully understood, but the observations agree other literature results.

There is ample room for further investigations. The sensors used in this study were heated in a tube furnace. In the future, it is aimed at adding a heater at the bottom side of the sensor to operate the sensor as a stand alone device. A miniaturization on a ceramic hot-plate (as shown in [34]) is another step for future research. In addition, the sensor mechanism has to be studied more in detail, with respect to a variation of the zeolite and the influence of the Cr_2_O_3_ layer, as well as with special respect to diffusion and reaction processes that occur when gases pass through and get inside the zeolite layer.

## Figures and Tables

**Table 1.** Results of the Cr electroplating.

**Final current during the electroplating process**	**Coloring of the Cr cover**	**Thickness of the Cr cover *d_Cr_***	**Thickness of the Cr_2_O_3_ cover *d***
10 mA	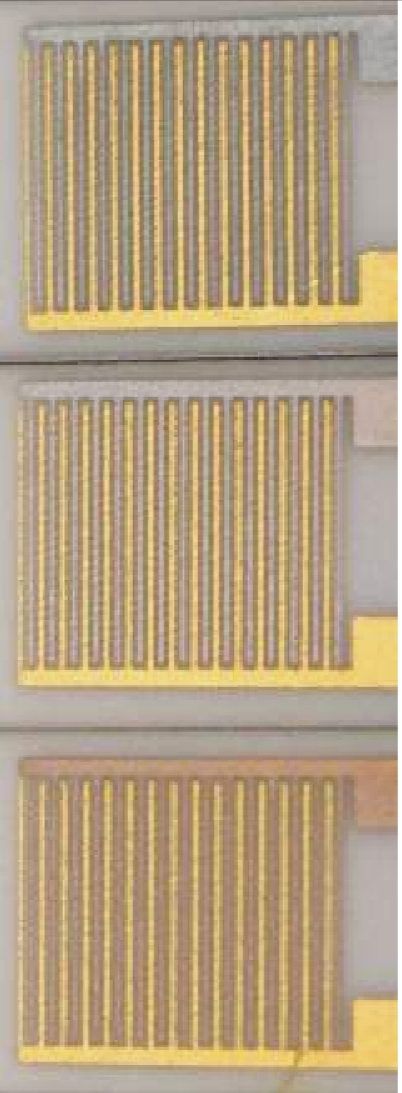	199 nm	251 nm
6 mA		119 nm	150 nm
3 mA		60 nm	75 nm

**Table 2.** Variation of sensor components.

**Pt content of the zeolite layer**	**Reduction route of the zeolite**	**Number of zeolite layers/thickness of zeolite layer**	**Thickness of the Cr_2_O_3_ layer *d***
1 wt%	NaBH_4_	1/25 μm	251 nm
2 wt%	H_2_	2/50 μm	150 nm
3 wt%		3/75 μm	75 nm
		4/100 μm	

## References

[1] Weitkamp J (2000). Zeolites and catalysis. Solid State Ionics.

[2] Kühl GH, Weitkamp J, Puppe L (1999). Modification of zeolites. Catalysis and Zeolites: Fundamentals and Applications.

[3] Raux S, Frobert A, Jeudy E (2009). Low temperature activity of euro4 diesel oxidation catalysts: Comprehensive material analyses and experimental evaluation of a representative panel. Top. Catal.

[4] Ghobarkar H, Schäf O, Guth U (1999). Zeolites—From kitchen to space. Prog. Solid State Chem.

[5] Löwenstein W (1954). The distribution of aluminum in the tetrahedra of silicates and aluminates. Am. Mineral.

[6] McCusker LB, Liebau F, Engelhardt G (2001). Nomenclature of structural and compositional characteristics of ordered microporous and mesoporous materials with inorganic hosts. Pure Appl. Chem.

[7] Puppe L (1986). Zeolithe—Eigenschaften und technische Anwendungen. Chem. unserer Zeit.

[8] Pina MP, Mallada R, Arruebo M, Urbiztondo M, Navascués N, de la Iglesia O, Santamaria J (2011). Zeolite films and membranes. Emerging applications. Microp. Mesop. Mater.

[9] Xu X, Wang J, Long Y (2006). Zeolite-based materials for gas sensors. Sensors.

[10] Sahner K, Hagen G, Schönauer D, Reiß S, Moos R (2008). Zeolites—Versatile materials for gas sensors. Solid State Ionics.

[11] Urbiztondo M, Pina P, Santamaría J, Valtchev V, Mintova S, Tsapatsis M (2009). Gas sensing with silicon-based nanoporous solids. Ordered Porous Solids: Recent Advances and Prospects.

[12] Vilaseca M, Coronas J, Cirera A, Cornet A, Morante J, Santamaría J (2003). Use of zeolite films to improve the selectivity of reactive gas sensors. Catal. Today.

[13] Alberti K, Fetting F (1994). Zeolites as sensitive materials for dielectric gas sensors. Sens. Actuat. B Chem.

[14] Plog C, Maunz W, Kurzweil P, Obermeier E, Scheibe C (1995). Combustion gas sensitivity of zeolite layers on thin-film capacitors. Sens. Actuat. B Chem.

[15] Hagen G, Dubbe A, Fischerauer G, Moos R (2006). Thick-film impedance based hydrocarbon detection based on chromium(III) oxide/zeolite interfaces. Sens. Actuat. B Chem.

[16] Franke ME, Simon U, Moos R, Knezevic A, Müller R, Plog C (2003). Development and working principle of an ammonia gas sensor based on a refined model for solvate supported proton transport in zeolites. Phys. Chem. Chem. Phys.

[17] Neumeier S, Echterhof T, Bölling R, Pfeifer H, Simon U (2008). Zeolite based trace humidity sensor for high temperature applications in hydrogen atmosphere. Sens. Actuat. B Chem.

[18] Urbiztondo M, Pellejero I, Rodriguez A, Pina MP, Santamaria J (2011). Zeolite-coated interdigital capacitors for humidity sensing. Sens. Actuat. B Chem.

[19] Simon U, Flesch U, Maunz W, Müller R, Plog C (1998). The effect of NH_3_ on the ionic conductivity of dehydrated zeolites Na beta and H beta. Microp. Mesop. Mater.

[20] Hagen G, Schulz A, Knörr M, Moos R (2007). Four-wire impedance spectroscopy on planar zeolite/chromium oxide based hydrocarbon gas sensors. Sensors.

[21] Dubbe A (2011). Integrated impedance based hydrocarbon gas sensors with Na-zeolite/Cr_2_O_3_ thin film interfaces: Electrochemical mechanism of the impedance changes. Phys. Status Solid. A.

[22] Näfe H (2007). Zeolite based hydrocarbon sensor-Re-interpretation of the principle of functioning. Electrochim. Acta.

[23] Dubbe A, Moos R (2006). Solid electrolyte hydrocarbon gas sensor using zeolite as auxiliary phase. Electrochem. Solid-State Lett.

[24] Dubbe A (2008). Influence of the sensitive zeolite material on the characteristics of a potentiometric hydrocarbon gas sensor. Solid State Ionics.

[25] Dubbe A, Wake M, Sadaoka Y (1997). Yttria/carbonate composite solid electrolytes for potentiometric CO_2_ sensors. Solid State Ionics.

[26] Cañizares P, de Lucas A, Valverde JL, Dorado F (1997). n-Butane Hydroisomerization over Pt/HZSM-5 Catalysts. Ind. Eng. Chem. Res.

[27] Dubbe A (2009). The effect of platinum clusters in the zeolite micropores of a zeolite-based potentiometric hydrocarbon gas sensor. Sens. Actuat. B Chem.

[28] Hagen G, Moos R (2011). Planar zeolite-based potentiometric gas sensors. Sens. Lett.

[29] Reiß S, Hagen G, Moos R (2008). Zeolite-based impedimetric gas sensor device in low-cost technology for hydrocarbon gas detection. Sensors.

[30] Tamási A, Niesz K, Pálinkó I, Guczi L, Kiricsi I (2002). Modifying the acidic properties of Pt-ZSM-5 and Pt-Y zeolites by appropriately varying reduction methods. Stud. Surf. Sci. Catal.

[31] Blase R (1994). Temperaturunabhängige Sauerstoffsensoren mit kurzer Einstellzeit auf der Basis von La_2_CuO_4+δ_-Dickschichten.

[32] Sahner K, Schönauer D, Kuchinke P, Moos R (2008). Zeolite cover layer for selectivity enhancement of p-type semiconducting hydrocarbon sensors. Sens. Actuat. B Chem.

[33] Sahner K, Schönauer D, Moos R, Matam M, Post ML (2006). Effect of electrodes and zeolite cover layer on hydrocarbon sensing with p-type perovskite SrTi_0.8_Fe_0.2_O_3-δ_ thick and thin films. J. Mater. Sci.

[34] Rettig F, Moos R (2004). Ceramic meso hot-plates for gas sensors. Sens. Actuat. B Chem.

